# High-Entropy Alloys for Advanced Nuclear Applications

**DOI:** 10.3390/e23010098

**Published:** 2021-01-11

**Authors:** Ed J. Pickering, Alexander W. Carruthers, Paul J. Barron, Simon C. Middleburgh, David E. J. Armstrong, Amy S. Gandy

**Affiliations:** 1 Department of Materials, University of Manchester, Manchester M13 9PL, UK; alexander.carruthers@manchester.ac.uk (A.W.C.); paul.barron@manchester.ac.uk (P.J.B.); 2 Henry Royce Institute, Manchester Hub Building, Manchester M13 9PL, UK; 3 Nuclear Futures Institute, Bangor University, Bangor LL57 2DG, UK; s.middleburgh@bangor.ac.uk; 4 Department of Materials, University of Oxford, Oxford OX2 6HT, UK; david.armstrong@materials.ox.ac.uk; 5 Department of Materials Science and Engineering, University of Sheffield, Sheffield S1 3JD, UK; a.gandy@sheffield.ac.uk

**Keywords:** high entropy alloys, nuclear fission, nuclear fusion, accident tolerant fuels, alloy design

## Abstract

The expanded compositional freedom afforded by high-entropy alloys (HEAs) represents a unique opportunity for the design of alloys for advanced nuclear applications, in particular for applications where current engineering alloys fall short. This review assesses the work done to date in the field of HEAs for nuclear applications, provides critical insight into the conclusions drawn, and highlights possibilities and challenges for future study. It is found that our understanding of the irradiation responses of HEAs remains in its infancy, and much work is needed in order for our knowledge of any single HEA system to match our understanding of conventional alloys such as austenitic steels. A number of studies have suggested that HEAs possess ‘special’ irradiation damage resistance, although some of the proposed mechanisms, such as those based on sluggish diffusion and lattice distortion, remain somewhat unconvincing (certainly in terms of being universally applicable to all HEAs). Nevertheless, there may be some mechanisms and effects that are uniquely different in HEAs when compared to more conventional alloys, such as the effect that their poor thermal conductivities have on the displacement cascade. Furthermore, the opportunity to tune the compositions of HEAs over a large range to optimise particular irradiation responses could be very powerful, even if the design process remains challenging.

## 1. Introduction

It will likely be difficult to commercially exploit high-entropy alloys (HEAs) in most engineering structural applications where steels, Al alloys or Mg alloys have a strong foothold, because HEAs are unlikely to be able to compete in terms of price and/or specific properties. For instance, HEAs designed for automotive applications must compete with steels that usually comprise very low concentrations of expensive elements, or Al alloys or Mg alloys that provide superior properties per kg at reasonable cost. However, there are a number of structural applications for which the pursuit of HEAs appears more attractive—these are the applications where our current suite of engineering alloys fail to function adequately in the demanding environments we wish to operate in, and it makes commercial sense to use more expensive or dense alloys if they are able to open up these new operational regimes. A number of such applications are associated with advanced nuclear power systems, and this review will explore HEA development for them. Specifically, it will focus on HEAs as engineering structural alloys for fusion power plants and Gen-IV fission power plants, and for accident-tolerant fuel (ATF) cladding for both Gen-III and Gen-IV fission. As highlighted by Zinkle and Was [1], the degradation effects at work in these next-generation applications are numerous and challenging.

The literature examining HEAs for nuclear applications remains relatively limited in size in comparison to that of the wider field. As of June 2020 (shortly before Scopus’ paper search function was removed) it was found that no more than 100 papers considering HEAs from a nuclear application standpoint had been published, with these typically focussing on alloy design and/or irradiation damage response. This compared to ∼4000 papers published in the HEA domain as a whole [2]. It seems, therefore, an opportune moment to summarise the state of this part of the HEA field and highlight possible directions for future study. The defect dynamics and irradiation performances of concentrated solid-solution alloys in comparison to conventional alloys were reviewed by Zhang et al. [3] in 2017, with a particular focus on the associated atomistic modelling. This review also examines HEA irradiation studies, but additionally assesses practical challenges associated with the design and implementation of HEAs in nuclear environments, such as those associated with element selection (alloy design).

This review begins by outlining the challenging service conditions that are associated with nuclear fission and fusion plants, and also highlights the restrictions that apply to our choice of alloying elements in such applications. It then moves on to assess the literature associated with HEAs for nuclear, specifically relating to their irradiation tolerance, before highlighting the key challenges associated with HEA development for advanced nuclear applications, as well as some important gaps in our knowledge that need addressing. Of course, for an HEA to be successful as a nuclear material it is likely to require a combination of properties including, for example, adequate processibility, strength, toughness, corrosion resistance, and so forth. This review will not comprehensively review each of these properties (e.g., it will not address the literature regarding the corrosion resistance of HEAs in specific environments in detail), but will highlight examples of such properties where appropriate.

In this review, our working definition of an HEA is based on composition only: an HEA is an alloy that comprises at least five components in concentrations between 5 and 35 at.%. Alloys that comprise fewer elements, but still high concentrations of each, are referred to as *concentrated alloys* for the sake of brevity. A distinction is made between these alloys and traditional engineering alloys, which are referred to as *conventional alloys* (even though they themselves might be concentrated).

## 2. Challenging Environments in Fission and Fusion

The challenging environments associated with next-generation fission and fusion are likely vary markedly according to the reactor design and the particular component of interest, but may involve extremes of temperature, corrosion, stress, and heat flux. Such conditions are also faced in other advanced engineering applications. However, there is further consideration that is of concern to nuclear design engineers—that of irradiation damage.

The irradiation damage caused in an alloy depends not only on the identity of the incident particle (i.e., whether electron, neutron, proton or heavy ion) and its energy, but also the characteristics of the material being irradiated (such as atomic weight, bond strength, crystal structure, stacking fault energy, composition and microstructure), as well as and the temperature of irradiation (homologous temperature, $T/T_{m}$). The irradiation damage of alloys is a topic of considerable complexity, and the intricacies are not the focus of this review. Readers are instead referred to comprehensive works elsewhere, for example, References [4,5]. However, a brief summary of the basic mechanism of irradiation damage is provided for background as follows, before the particular environments associated with next-generation nuclear applications are explored.

Considering the case in which an energetic neutron (e.g., with energy of 1 MeV or more) is incident on an alloy, the collision between the neutron and an atom may result in the atom being displaced from its lattice site with considerable kinetic energy. This primary knock-on atom (PKA) possesses sufficient energy to go on to collide and displace other atoms, which in turn collide with others, and so on, creating a *displacement cascade*. The result is a large number of displacements and the formation of corresponding Frenkel pairs (FPs)—vacancy + interstitial pairs—with the interstitial atoms often spatially separated from their corresponding vacancy. These FPs are the primary damage caused to the material during irradiation, and it is the evolution of this damage that results in all other irradiation-induced damage phenemona. There is also an increase in temperature locally shortly after these FPs are created, a *thermal spike*, as the energy from the cascade is dissipated. In the short-term (typically a few picoseconds following the damage cascade, which itself is typically only one or two picoseconds in duration) the interstitials created can diffuse to either recombine with vacancies, be absorbed at sinks such as grain boundaries, or group together to form small clusters. Interstitials are typically far more mobile than vacancies, but at at high homologous temperatures (typically ($T/T_{m}$ > 0.2), vacancies can do the same. In the long-term (seconds-years), the movement of these FPs continues and leads to the formation of extended defects and long-range damage phenomena. At low homologous temperatures ($T/T_{m}<0.2$), interstitials and interstitial clusters move and combine to form extended defects such as dislocation loops. At higher homologous temperatures ($T/T_{m}$ = 0.2–0.6), the movement of both interstitials and vacancies can result in void swelling, radiation-induced segregation (RIS), and radiation-induced phase transformations and precipitation (RIP) (Note that the affect of temperature is also often explained in reference to five distinct ‘stages’ of damage recovery. These have not been described here for reasons of brevity, but readers are referred to summaries in, for example, Reference [5].). All of these can change the mechanical properties of an alloy dramatically, almost always degrading them, and leaving their properties outside of certified ranges. The damage evolution is ultimately a function of defect production, recombination, absorption, and clustering, which are all strongly influenced by temperature and are also affected by alloy composition and microstructure (amongst other factors). The resulting degradation is also a function of the total ballistic damage applied to a material, typically measured in units of displacements per atom (dpa), as well as the damage rate (determined by the irradiation flux).

### 2.1. Nuclear Fusion

**Figure 1:**
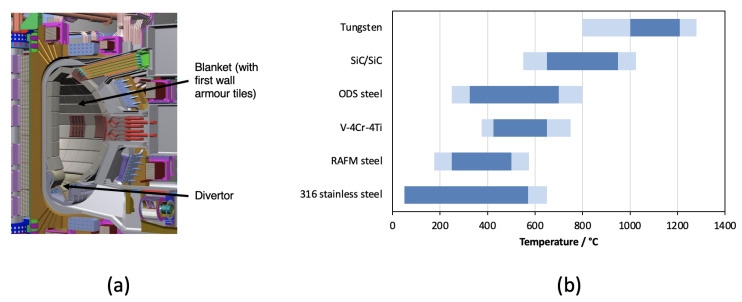
(**a**) Cross section of the ITER tokomak, showing location of first wall, blanket and divertor structures. (**b**) Operating temperature windows (based on radiation damage and thermal creep considerations) for selected fusion candidate materials. The light shaded bands on either side of the dark bands represent the uncertainties in the temperature limits. This figure is a modified version of that first presented in Reference [8].

The conditions inside future commercial fusion reactors will be the most extreme of any next-generation nuclear application. A high-flux of 14.1 MeV neutrons will lead to damage levels of over 100 dpa in some components, which may also experience high temperatures, high levels of internal H and He generation (via transmutation), erosion and implantation from the plasma, corrosion from coolants, strong magnetic fields, and extreme heat loads (particularly during transient plasma events). Three regions of a fusion tokamak that experience the most extreme conditions are highlighted in Figure 1a, along with some candidate materials and their nominal service temperatures in Figure 1b. The extreme conditions found in the divertor region of a tokamak, where the plasma exhaust is directed, most likely restrict candidate materials to pure tungsten (or alloys comprising predominantly of tungsten) owing to the high melting point required. The blanket, which will house systems to extract heat and breed tritium to refuel the reactor, will not see such extreme temperatures, and neither should the first wall ‘armour’, which shields the blanket from the plasma (at least not under nominal steady-state operating conditions). Hence, there is scope to use alloys comprising lower-melting point elements than tungsten. Nevertheless, the level of irradiation damage experienced by these structures will still be severe, and they will operate at hundreds of degree celsius.

None of the candidate materials listed in Figure 1b is likely to be the perfect fit for the fusion first wall or blanket. For instance, oxide-dispersion-strength (ODS) steels are notoriously difficult to manufacture in bulk, and the lower operation temperature limit of vanadium alloys is likely to be 300 °C or above [6]. The creep performances of reduced-activation ferritic-martensitic steels (RAFM steels, e.g., EUROFER 97) tend to be worse than the best conventional power plant steels (e.g., P91, P92) [7]. Also, it is beneficial to note that none of these materials has been assessed in a truly representative service environment, because we do not have a source of 14.1 MeV neutrons that is capable of delivering the damage that would be encountered. There is a real possibility that candidate conventional alloys will perform poorly, and we will need to search for alternatives.

It is interesting to note that the properties of irradiated HEAs may be of relevance to the fusion community regardless of whether or not they are used to fabricate reactors, since high levels of transmutation may create compositionally-complex alloys from conventional alloy starting materials, as has already been noted in fission products [9,10].

### 2.2. Gen-IV Fission

There are a number of different designs based around the use of different high-temperature coolants—for example, liquid sodium, liquid lead, helium and molten salts are common examples. The damage levels expected in the components of such reactors are typically significantly higher than in conventional pressurised water fission plants, and the core operating temperatures are much higher (typically > 600 °C) [11]. As highlighted by Zinkle and Was [1,12], there are considerable challenges associated with compatibilities with the coolants being used (i.e., high-temperature corrosion), but perhaps the most serious challenge is posed by material degradation from high-temperature irradiation to very high doses. Excellent void swelling resistance and microstructural stability will be essential. Many of the candidate structural materials are also candidates for fusion reactors, such as ODS steels and refractory alloys, and have been selected on the basis of their good high-temperature mechanical properties. Nevertheless, there remain the same challenges to their implementation as discussed in the section above, with many related to processibility and low-temperature brittle behaviour.

### 2.3. Accident-Tolerant Fuel (ATF) Cladding

The catastrophic explosions that took place during the Fukishima disaster in 2011 were due to the combustion of hydrogen that had been produced by the high-temperature oxidation of the Zr alloy fuel cladding by steam [13] (Zr${}_{\left(s\right)}$ + H${}_{2}$O${}_{\left(g\right)}$→ ZrO${}_{2}$${}_{\left(s\right)}$ + H${}_{2}$${}_{\left(g\right)}$). Zr alloy tubes are used in most light-water fission reactors to house the fissile fuel, and so there is considerable impetus to explore options to eliminate the chances of such failures in the future. This has led to a significant body of research in the past decade concerning the development of accident-tolerant fuel claddings, which resist high-temperature oxidation in steam [14].

As well as exposure to high-temperature steam in an accident scenario, ATF cladding materials will also have to deal with prolonged exposure to irradiation damage and corrosion during normal service conditions. There may also be additional demands placed on them to facilitate fuel reprocessing. A number of promising candidate ATF cladding systems have been identified and continue to be explored. Some of these involve the modification of Zr alloy tubes with coatings, such a chromium, whilst others involve the development of entirely new tubing systems, such as those based on silicon carbide fibre-reinforced silicon carbide matrix composites [14]. At present, there is no clear winner, and each has its own challenges. There remains an opportunity to develop solutions based on more exotic alloy compositions, albeit with a restricted elemental palette, as discussed below.

## 3. Desired Properties and Element Restrictions

Given the conditions described above, alloys for advanced nuclear applications will require some combination of—adequate strength and ductility, creep resistance, irradiation resistance, corrosion resistance, and microstructural stability, and all across a range of temperatures and/or at high temperature. There are also properties that are specific to particular applications, for example, ATF cladding alloys must be sufficiently processible to be made into thin-walled tubes, and alloys for fusion plasma-facing components must resist H embrittlement and retention.

The desired properties place restrictions on the compositions and microstructures of the alloys that we use, much as they would in any other engineering application. However, there are some specific requirements that place fundamental restrictions on the use of *individual elements* themselves in a way that is not commonly found in other engineering applications (notwithstanding the universal restriction of price per kg). This has immediate implications for HEA design.

### 3.1. Activation Issues

For both fission and fusion plants, the radioactivity caused by the activation of elements by neutron irradiation is a concern. The concern is comparatively less serious in fission plants, since so much hazardous waste is produced unavoidably from the fuel. Nevertheless, the use of activating elements in some fission plant components is being actively avoided to minimise the exposures of plant operators and decommissioners, and minimise the amount of legacy waste. For instance, there has been a concerted effort to move away from Co-containing hard-facing materials in primary circuit water pumps, since Co finds its way into the primary water, activates, and can contaminate the whole coolant circuit.

Elemental activation with respect to fusion power is an even greater and more pervasive concern. Implicit in the favourable political outlook that has been afforded to fusion power is the notion that plants would not produce any long-lived hazardous radioactive waste. This is seen as critical to fusion power being socially acceptable and commercially successful. Elements used should ideally decay to low level waste level or below in 100 years or less following removal from a reactor (and even then, impurity elements may make it difficult to meet the desired low activation levels).

**Figure 2:**
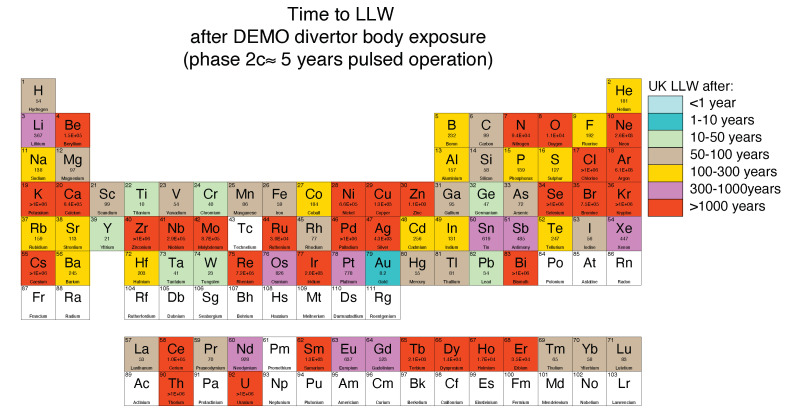
Periodic table of the calculated time period that each element would require to decay to UK low level waste limits (<4 MBq kg${}^{-1}$ alpha radiation and <12 MBq kg${}^{-1}$ combined gamma and beta radiation) following exposure inside a DEMO fusion reactor. The component considered is the divertor over a time period equivalent to 5 years of pulsed operation. Reproduced from Gilbert et al. [15].

The particular activation that an element will undergo depends on the neutron energy spectrum and flux it faces (and hence its place in a reactor relative to the neutron source). Figure 2, produced by Gilbert et al. [15] using the FISPACT-II neutronics code, indicates how long elements would take to decay to be classed as low level waste in the UK following exposure to the fusion neutron spectrum in the divertor of a hypothetical fusion reactor. This gives a good indication as to activation inside a fusion reactor. We can see that many of the elements that we use in many engineering alloys are precluded, including Ni, Zr, Nb and Mo.

A number of studies have considered HEAs based on low-activation elements like Ti, V, Cr, Mn, Fe, Ta and W [16,17,18,19,20,21,22,23,24], although their development remains in its infancy. Despite increasing the ease of alloy design efforts (there are fewer elements and combinations to choose from), there are some serious challenges associated with the use of these elements to form HEAs, as will be discussed later in Section 5.1.2 and Section 5.2. Whilst these element are relatively passive in the long-term, some remain highly active in the short-term, in particular Mn. Such elements may present problems for maintenance operations, and their decay heat may be a considerable concern in loss-of-coolant accidents [25,26]. Nevertheless, there is also the possibility (even if it remains far-fetched) that this short-term decay heat be utilised positively, for example, for smoothing pulsed-operation power cycles, or for in-situ annealing of damage.

### 3.2. Neutron Cross Section

It is imperative that any ATF cladding solutions retain a low neutron cross section, such that neutrons are able to escape the fuel rods to have their energies moderated to suitable levels for further fission. This is one of the principal reasons why Zr was chosen for fuel cladding applications, and why it has been so successful. Using elements with low neutron cross sections is also important in fusion reactors, where neutrons from the plasma must pass through the plasma-facing structures to collide with lithium-6, triggering a transmutation that produces tritium. A fusion reactor must be self sufficient in terms of tritium in order to be viable, and there are only a limited number of neutrons that are produced by each fusion event (one for each tritium atom consumed). Hence, an efficiency of >1 breeding events per neutron produced is required to maintain the reaction (accounting for losses). Neutrons must be able to penetrate to the tritium breeding site.

A useful lookup tool for the neutron scattering and capture cross sections for elements as a function of neutron energy is the TENDL nuclear data library [27]. A number of studies have used low neutron cross section elements to design HEAs for ATF cladding, either as bulk alloys [28,29,30,31] or as coatings for Zr alloys [32,33,34,35,36]. Preliminary results of the HEA coatings has suggested better wear and corrosion performance than uncoated Zr, and authors have claimed they are good candidates for ATF cladding [33,34]. However, HEAs have not been compared to other more conventional candidate coating systems, and the properties are very dependent on composition [36].

### 3.3. BCC vs. FCC

It is a commonly held view that body-centred cubic (BCC) metals exhibit superior resistance to void swelling than do face-centred cubic (FCC) metals. Much of this sentiment has arisen from the extensive research that has shown ferritic steels to exhibit markedly lower void swelling than their austenitic counterparts under the same irradiation conditions [37]. This has has primarily been attributed to a delay in the onset of swelling, and it has been suggested that this delay is due to the increased difficulty of void nucleation in ferritic alloys [37,38], but the mechanisms remain unclear. Interestingly, there are indications that BCC metals are also superior to FCC metals in terms of their primary defect production and defect evolution behaviours [39,40,41]. Hexagonally close-packed (HCP) metals are generally not favoured owing to their anisotropic responses.

Nevertheless, it has been found that for some BCC alloy compositions, void swelling can be very significant at high damage levels, and the resistance (or lack of) can be very dependent on composition [42,43,44]. Once the onset of swelling is passed, they can swell at a high rate, and the presence of H and He shortens the onset [37]. It also appears that when they do undergo void swelling, the peak in their swelling is often found at comparatively low homologous temperatures in comparison to FCC metals, and their temperature dependence of swelling can be quite different [5]. It is well known that BCC metals tend to have higher atomic diffusivities in comparison to FCC metals at the same homologous temperature [45], so it should not be surprising that processes that depend on diffusion are apparently accelerated in BCC alloys. Their reported improved primary defect behaviour in comparison to FCC and HCP metals is still not well understood, and could well be attributed to compositional rather than structural effects [39]. Overall, BCC alloys remain a preferred option (not least for the activation reasons discussed above), but care must be taken in generalising their superiority, and the effects of composition can predominate [37]. Unfortunately, there is a lack of systematic studies comparing the responses of FCC, BCC and HCP metals under the same irradiation conditions.

## 4. The Potential Suitability of HEAs—Irradiation Resistant?

It is clear from the discussion in Section 2 that nuclear HEAs will need to possess the good mechanical and environmental properties that most engineering alloys are required to. Such properties are not addressed in this review, and have been the subject of a significant number of HEA studies that are summarised in a number of reviews, for example, Reference [46]. Here we focus on the irradiation response specifically.

A number of suggestions were made in early studies about the special resistance of HEAs to irradiation damage, these include (i) higher resistance to irradiation defect formation [47,48,49,50], (ii) lower void swelling in comparison to conventional alloys [51,52,53], (iii) higher microstructural stability (usually phase stability) than conventional alloys under irradiation [49,51,52,54,55], and (iv) limited irradiation hardening [51]. Such generalisations have been less common in more recent work, but there is still an underlying sentiment in many reports that HEAs have special irradiation resistance.

The following sections address three effects that have been commonly cited with respect to the irradiation response of concentrated alloys: (i) the effect of reduced thermal conductivity on cascade dynamics, (ii) the effect of sluggish diffusion on damage accumulation, and (iii) the effect of defect formation energies on damage accumulation. Observations of various irradiation-induced phenomena in concentrated alloys (e.g., void swelling) are included where appropriate. Following this, discussions of phase stability and hardening behaviour under irradiation are summarised. There is a focus on studies examining concentrated additions to alloys (e.g., binary ≥ 10 at.% solute), particularly those building towards our understanding of HEA behaviours over recent years.

### 4.1. Thermal Conductivity and the Displacement Cascade

Many authors have suggested that the lower thermal conductivity afforded by the compositional complexity of concentrated alloys leads to more limited energy dissipation immediately following the displacement cascade [3,50,56,57,58,59]. Instead of being dissipated over a wide volume, the energy from the PKA is more localised and concentrated owing to increased phonon scattering, meaning a longer and more spatially concentrated thermal spike. This should give more time and energy for FP defects to recombine. The results of some room temperature molecular dynamics (MD) studies, which compared defect generation after a cascade in NiFe and NiCo binaries to pure Ni, have supported this hypothesis. They have found that in NiFe, which has a significantly lower thermal conductivity than both pure Ni and NiCo, there are significantly fewer stable FP defects present (inclusive of both separated and clustered defects) after a cascade at relatively high ballistic PKA energies (≥10 keV) [56,58,60]. Fewer defects are also produced in NiCo, which has a thermal conductivity that is marginally lower than Ni at room temperature, although this was only found to be the case at very high ballistic energies [58]. MD simulations of NiCoCrFe found fewer FP defects were produced in comparison to pure Ni across a range of PKA energies, with the effect being more pronounced at higher energy. This result was similarly attributed to the effect of the concentrated alloy’s lower thermal conductivity on prolonging the thermal spike [59].

Nevertheless, the picture is not a clear one. For instance, the results of Beland et al. [58] suggested that the effect of thermal conductivity may only be partially responsible for the reduced number of FPs created in the Ni binary alloys, and only at energies below 20 keV [58]. There are also indications that effect is also likely to wash out at high temperature [61], where phonon scattering by thermal vibrations is enhanced. Some work has found no significant difference in the number of surviving FPs between pure metals and alloys even at room temperature. For instance, at 5 keV, simulations of Ni${}_{0.8}$Fe${}_{0.2}$ and Ni${}_{0.8}$Cr${}_{0.2}$ showed no significant difference in the number of FPs produced per cascade [57]. In the Fe-Cr system, simulations across a range of PKA energies have found no difference between concentrated Fe-Cr and Fe-Ni alloys and Fe [62], despite the addition of Cr to Fe decreasing thermal conductivity [63]. Some studies have even found the reverse trend [64,65] (i.e., more damage is created per cascade in concentrated alloys).

Somewhat in contradiction to the above thermal conductivity based argument, others have suggested that the initial damage caused by the cascade is *enhanced* in HEAs by high inter-atomic stress levels, owing to atomic size differences [47,55,66,67]. It has been argued that these stresses facilitate easier amorphisation of HEAs by the displacement cascade, in a similar way to the effect of increasing temperature has on amorphisation on melting (i.e., increased thermal vibrations aides melting). However, the same authors suggest that rapid recrystallisation can follow this amorphisation, wiping out any irradiation defects. They proposed, therefore, that HEAs are “self-healing” [47,55,66,67]. This idea, that an alloy with a highly-strained lattice is more intrinsically resistant to irradiation, is at odds to what would normally be considered best for alloy stability.

### 4.2. Sluggish Defect Mobilities

Many studies have indicated that defect mobilities may play a significant role in the different irradiation responses of concentrated alloys in comparison to pure metals and conventional alloys, with respect to both short-term damage (initial defect clustering and damage accumulation) and longer-term damage (voiding and radiation-induced segregation). The mechanisms proposed are predominantly based around the idea of *slower* or *sluggish* defect mobilities in more compositionally complex alloys.

#### 4.2.1. Short-Term Damage

Many studies have found that defect clusters are smaller and more numerous in concentrated Ni binaries than in pure Ni at room temperature, both via experimental measurements and simulation [56,57,68,69,70,71,72,73,74]. A general trend towards smaller and more numerous defect clusters has also been found with increasing atomic size difference between the solute atom and Ni at higher temperatures [75], and also on increasing the number of alloying additions (from concentrated binaries to concentrated quinaries) in alloys based around the CrFeCoNi family both at room and higher temperatures [59,76,77]. The same trend has also been found when concentrated alloys have been compared to traditional Fe-Cr-Ni austenitic alloys after irradiation under similar conditions [78,79]. These findings have most often been attributed to slower defect mobilities in concentrated alloys, which limits the extent to which they are able to find clusters and clusters are able to coarsen [56,57,68,69,72,75,76,78,79]. Models have also suggested a greater fraction of stable interstitial defects end up in clusters in pure Ni than in Ni binaries [57,58,68,74], which has been taken to support the idea that defect mobilities are slower in more compositionally-complex alloys (HEAs).

**Figure 3:**
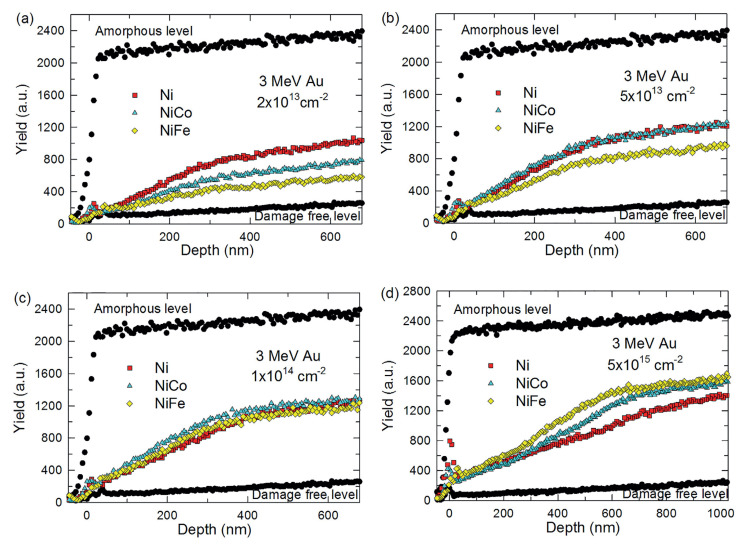
Rutherford backscattering channeling spectra of Ni, NiCo and NiFe, irradiated with 3 MeV Au ions to the fluences ranging from 2 × 10${}^{13}$ (**a**), 5 × 10${}^{13}$ (**b**), 1 × 10${}^{14}$ (**c**) to 5 × 10${}^{15}$ (**d**) ions cm${}^{-2}$ Reproduced from Reference [80].

Additionally, comparative studies of pure Ni and Ni binaries at room temperature have found that damage accumulates faster in pure Ni and saturates earlier, whilst damage in the alloys accumulates more slowly, but it may eventually peak at a higher level at higher doses [70,80]. See, for example, the results of Rutherford backscattering (RBS) measurements in Figure 3 [80]. The accumulation in ternary and higher-order alloys seems to be even more sluggish than the binaries [3,76,81]. This trend has been rationalised by suggesting that although fewer defects are created by each cascade in the concentrated alloys (owing to the cascade kinetic effects outlined above), reduced defect mobilities provides fewer opportunities for inter-cascade annealing [56,58,69], eventually leading to a higher level of damage at saturation. Observations of damage at deeper depths in pure Ni in comparison to binaries have also been attributed to slower interstitial diffusion [70,80].

It should also be noted that trends in damage accumulation seem to depend not only on the particular composition examined, but also the irradiation conditions (total dpa, ballistic energy and species, temperature, etc.), for example, see Reference [82] for the effect of temperature on damage accumulation and depth in NiFe. Studies have sometimes described trends that apparently contradict those reported by others. For instance, works examining defect generation using in-situ irradiation of thin films have reported a lower density of defects in more concentrated alloys, and that these were larger in size than in pure Ni [81,83,84].

Granberg et al. [64,65,74] have highlighted the potential importance of the mobility of dislocation defects on irradiation damage accumulation. They argue, along with others [77], that dislocation motion may be slower owing to the lattice distortion effect in concentrated alloys, and that this may also contribute to slower damage accumulation in them. It should be noted that the trend in damage accumulation they were attempting to explain was somewhat conflicting with the trend described above (i.e., they were explaining why the initial damage accumulation may be faster in the concentrated alloys than in pure Ni, and why the saturated damage may be higher in pure Ni, which is in conflict to the Figure 3 trend). Nevertheless, a change in the motion of dislocation defects should influence the irradiation response of concentrated alloys. Indeed, experimental investigations have indicated that loop migration is more difficult in more compositionally-complex concentrated alloys based around CrFeCoNi [83], and lattice distortion measurements have supported these trends [77].

#### 4.2.2. Longer-Term Damage

A number of studies have measured the irradiation void swelling in concentrated alloys, including HEAs, to be significantly lower than in pure metals, often by over an order of magnitude for the same damage level [3,52,85,86,87,88]. The trend has been reported to be real even when the suppression of voiding by injected interstitials is accounted for [87], and follows trends reported from the 1970s onwards in Ni-Cu alloys [89,90,91,92]. Some have found little evidence of void formation under conditions where they would typically be expected in conventional alloys [67,78,93,94], although most others have seen voiding, but at a suppressed level [75,80,86,87,88,95]. For instance, significant void swelling has been observed in CrFeCoNi, but only at high damage doses, 86–250 dpa. Between 16 and 54 dpa the swelling observed was negligible, and far lower than in pure Ni [88]. There is an apparent effect of the atomic size difference between atoms on the swelling behaviour observed [75,95], and it is also clear that additions of certain elements can increase void swelling [54].

Apparent increases in void swelling resistance in concentrated alloys have most often been attributed to the slower migration of defects and defect clusters, and in particular the slowing of interstitials and interstitial clusters. Vacancies are mobile at high temperatures, but interstitials still remain much faster (as they are at room temperature). Hence, interstitials are able to diffuse rapidly away from the damaged region to sinks like grain boundaries. This leaves an excess of vacancies that precipitate as voids. However, if the mobilities of interstitials can be reduced such that they are more similar to vacancies mobilities, they can instead persist near the original cascade for long enough to recombine with vacancies and suppress void swelling (and other clustering). A number of studies have suggested (or simulated, e.g., using MD models [86]) that interstitials and interstitial clusters are significantly less mobile in concentrated Ni alloys than they are in pure Ni, and that their mobilities are brought more in line with vacancy mobilities, which they have used to explain the voiding resistance observed [86,87,96,97].

With regards to He bubble formation, it has been claimed that concentrated alloys have a higher resistance when compared to pure metals and some steels [79,98,99,100], and this has been attributed to sluggish diffusion of interstitials as highlighted above—if He atoms move through vacancies, then fewer, slower vacancies should improve resistance to He bubble formation, in addition to improving resistance to void swelling. This is somewhat at odds with older investigations that suggest more complex behaviour [101].

There have been a number of studies that have suggested that radiation-induced segregation (to both grain boundaries and dislocation loops) can be significantly suppressed with increasing number of elements in concentrated alloys [3,76,78,84]. This trend has also been attributed to the sluggish diffusion effect. The particular segregation behaviour appears to be composition-dependent [3,76,84], as is well known in conventional alloys [4,102,103].

#### 4.2.3. Mechanisms Associated with Reduced Mobilities

**Figure 4:**
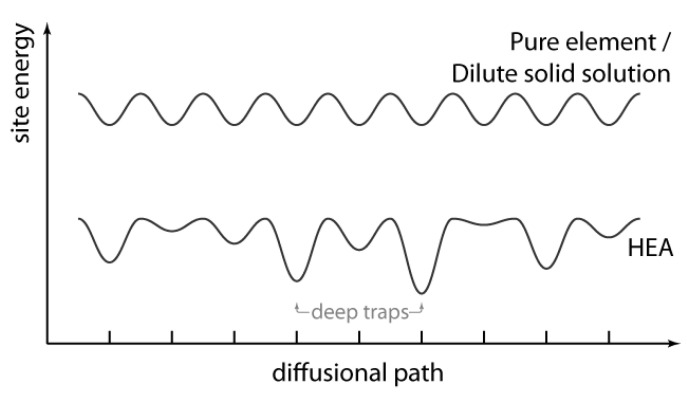
Schematic representation of the proposed difference in lattice potential energy profile along an atomic diffusion path in a pure element or dilute solid solution (**top**) and an high-entropy alloy (HEA) lattice (**bottom**). Note it is assumed that the distance between atomic sites is constant. Reproduced from Reference [108]. A similar schematic for the HEA energy profile was given in Reference [105].

As stated above, the reduced mobilities of defects (which can be mostly equated to sluggish atomic diffusion) has often been cited as a reason for the improved irradiation properties of concentrated alloys in comparison to pure alloys. Discussions of the mechanism by which mobilities are reduced have usually been based around the same ideas as those in the wider HEA literature—that is, that the variable chemical environments found in compositionally-complex alloys lead to a distribution in migration energies, and that some sites more effectively trap diffusing species and hence reduce overall mobility [104,105]. See Figure 4 for an illustration of this. The results of atomistic simulations suggest this is indeed the case—there is a distribution of migration energies in concentrated alloys, and on average the migration energies are higher for interstitials and interstitial clusters in Ni alloys versus pure Ni [3,69,86,96,97,106]. The trend for vacancies is less clear and may even be the reverse (i.e., lower migration energies) [87,97,107], which supports the idea that void swelling in concentrated alloys may be suppressed owing to the mobility of interstitials and vacancies being more similar than in pure metals.

Closely linked to this, lattice distortion has also been cited as a potential source of higher defect migration energy barriers in concentrated alloys, including of dislocations [3,68,74,75,77,78,86,95,98,99,109]. A distorted lattice, it is argued, should be harder to move through, and presumably produces the same site-to-site variation in migration barriers schematised in Figure 4. A difference in atomic size between alloying elements should effect the level of distortion present. The work of Yang et al. [75,95] suggests that this is the case and that different interstitial and vacancy trapping forces can arise depending on the atomic size difference. For instance, the inclusion of larger atoms should increase the distortion and vacancies are likely to be more strongly bound to them, thereby suppressing void growth more effectively [75,95].

Some studies have suggested trends in RIS (enrichment vs. depletion) in concentrated alloys can be rationalised by considering atomic size differences, since atoms diffuse according to local stress field [76,84,110]. However, this phenomena is well known in conventional alloys and the trends appear to be no different in concentrated alloys.

The results of simulations have suggested that a change in the geometries of defects and their migration paths may also be a cause of reduced interstitial mobilities, and resulting improved void suppression, in concentrated alloys [86,106]. For instance, it has been found that the 1D diffusion of interstitial clusters dominates in Ni, but in alloys sluggish 3D diffusion of clusters can be more prevalent, leading to reduced mobility [86].

The question then remains—is there evidence that defect migration in HEAs and high-order concentrated alloys is *abnormally* sluggish? In answer to this, the recent comprehensive review of thermally-induced diffusion in HEAs by Dąbrowa and Danielewski [111] concluded there is in fact *no conclusive experimental evidence* that diffusion is sluggish in HEAs. This is in agreement with other reviews that have pointed out that HEA diffusion does not appear to be anomalously slow in comparison to more conventional alloys [108,112]. Of course, comparing diffusion in standard thermal-only experiments is likely to be quite different to the diffusion that can occur during irradiation damage. For instance, species that would normally only be considered to diffuse substitutionally instead diffuse interstitially, and damage is caused by both interstitials and vacancies, which may be differently affected by alloy compositional complexity. Such distinctions from thermal diffusion may indeed lead to truly different behaviour in HEAs in comparison to conventional alloys. Nevertheless, the evidence for this is lacking and there are very few studies that compare irradiation diffusion to conventional alloys. One such study, by Chen et al. [113], compared microstructural evolution in HEAs to conventional alloys under irradiation and concluded that the effect of sluggish diffusion was insignificant.

With regards to lattice distortion, studies performing and reviewing measurements of lattice distortion of single phase HEAs have not found their lattices to be abnormally distorted [114,115], and this remains a topic of considerable contention. Although it is logical that the atomic size difference between elements should change defect migration behaviours, there is no reason to suppose the effect will manifest itself any differently in a highly-concentrated multicomponent alloys (i.e., an HEA) than in a simple binary alloy or a conventional alloy.

If differences are found between the diffusion behaviours of HEAs and more conventional alloys during irradiation, care must be taken to distinguish differences in mobility due to changes in defect migration energies (i.e., what is described above) and changes in defect *formation* energies. When new defect geometries are created, formation energies must be accounted for, and may predominate subsequent migration behaviour.

### 4.3. Defect Formation and Binding Energies

It has been highlighted by many authors that a change in the formation and/or binding energies of defects is also likely to have an effect on the irradiation responses of concentrated alloys. For many of the observations that were summarised above, authors have also attributed the behaviour to these energies in their explanations. For instance, the change in clustering behaviour, to smaller and more numerous defects in alloys, has been attributed to a change in the extended defect energies [56,58,68].

A number of atomistic modelling studies have found that, owing to the changeable local chemical environment in HEAs and other concentrated alloys, that there is a distribution of interstitial and vacancy formation energies [96,97,106,116,117]. There seems to be a general finding that vacancy formation energies are higher in concentrated alloys in comparison to pure Ni, and that this has been cited as another reason, in addition to sluggish diffusion, for increased void suppression [97,117]. It should be noted, however, that in other studies the trend is less clear, and Sugita et al. [107] recently found little difference in vacancy formation energy in CrFeCoNiMn in comparison to pure Ni [107].

Formation energetics could also influence the nature of the defects being generated in concentrated alloys versus pure alloys, and that this can then have a knock-on effect on the mobilities of those defects. For instance, dumbbells need to first form before they migrate, and the formation energy has been found to change depending on the composition [3,96,97], thereby influencing the prevalence of each.

Studies have often inferred lattice strain or inter-atomic stresses as part of their defect formation energy discussions, and it has been argued that these may destabilise defects in concentrated alloys, leading to more rapid recombination [47,48,49,55,66,97]. It is certain that local lattice strains caused by defects can help destabilise them (in much the same way as solute size differences can be seen to drive precipitation in alloys according to the Hume-Rothery rules [118,119]), but, as discussed above, abnormally high lattice strains or inter-atomic stresses are yet to be measured conclusively in HEAs (certainly not in HEAs that are microstructurally stable).

#### A Complex Picture

The energetics of irradiation damage phenomena are complex and there is unlikely to be one simple answer to explain any different behaviours observed in HEAs and other concentrated alloys. Taking the example of void swelling, extensive studies of swelling in austenitic stainless steels have shown that changes in composition can have a significant effect on the onset of rapid swelling, owing to changes in defect migration rates and formation energies. However, there are also many other factors that can play a role in swelling behaviour, such as the removal of elements by precipitation, changes in defect sink populations or biases due to composition, and changes in shear modulus the due to composition [102,120]. It is well known that void swelling tends to exhibit two distinct regimes—the transient regime, where the rate of swelling increases, and a steady-state regime, where the swelling rate is relatively constant. Each of these are differently affected by the factors mentioned above [102]. There have been only limited investigations of such behaviours in HEAs or concentrated alloys to date [88].

The complexities of void nucleation and growth in concentrated alloys have been illustrated by investigations of void distribution through the depths of irradiated layers. Voids in pure Ni and some binaries have been found to be significantly larger and more evenly-distributed through the irradiated depth than those in higher-order alloys, which have tended to show fewer smaller voids [3,80,85,86,87,91]. The size, density and distribution have been found to be dependent on composition, with an apparent effect of the atomic size difference between the atoms present [75,95]. Recently, Fan et al. [121] have suggested that irradiation-induced preferential diffusion can lead to changes in the concentration of elements through the depths of irradiated layers, which in turn can alter the spatial evolution of voids through them. Some studies have also claimed to observe voiding to be located predominantly outside the ion-irradiated region [86,87,88,95], which is unlike the damage seen in conventional alloys. To reach this conclusion, these studies needed to assume that SRIM predictions of damage in the concentrated alloys were sufficiently accurate.

### 4.4. Phase Stability and Hardening Resistance

A number of studies have found that some HEAs possess good phase stability under irradiation [49,51,52,54,55,67,93,94,95,110,122,123,124]. This behaviour has been attributed to a number of factors. Significantly reduced thermal conductivity leading to less damage as per the argument described in Section 4.1 has been invoked [54], as well as reduced defect mobilities or higher defect formation energies [54,122]. Entropic stabilisation has also been cited as a possible stabilisation mechanism [54,122].

Other HEAs, however, have been found to exhibit phase changes on irradiation, with some exhibiting extensive microstructural evolution, especially precipitation [21,22,84,113,122,125,126,127,128]. Owing to the propensity of most HEAs to decompose during thermal ageing [108,112], it is not always clear if the HEAs in these studies would have decomposed simply under thermal annealing, since many do not make a suitable comparison. However, some works have compared irradiated material with non-irradiated material exposed to the same thermal conditions (including longer thermal exposures). For instance, a recent study of phase stability in Al${}_{0.3}$CoCrFeNi found precipitation in both irradiated and non-irradiated areas, but that the particular effect of irradiation on the precipitation behaviour differed according to the temperature. At lower temperatures, ballistic mixing suppressed precipitation in irradiated areas in comparison to non-irradiated regions, but radiation-enhanced diffusion was found to enhance it at higher temperatures [127]. In a related alloy, Al${}_{0.12}$NiCoFeCr, Kombaiah et al. [126] showed that irradiation led to decomposition of the single phase solid solution, whereas longer thermal exposure at the irradiation temperature (500 °C) did not. Interestingly, they still concluded this was likely a case of radiation-enhanced precipitation (specifically, radiation-enhanced diffusion), rather than radiation-induced precipitation—that is, that the precipitates were likely to be stable phases under equilibrium conditions, and the precipitation was accelerated by enhanced diffusion. In a more likely case of irradiation-induced phase change, a sigma to BCC transition at room temperature has been observed in some alloys, which is very unlikely to have been favourable in the non-irradiated state [21,55].

Overall, mirroring trends in thermal stability found in the wider HEA literature [108], there is little evidence that HEAs exhibit enhanced phase stabilities in comparison to conventional alloys when they are irradiated.

**Figure 5:**
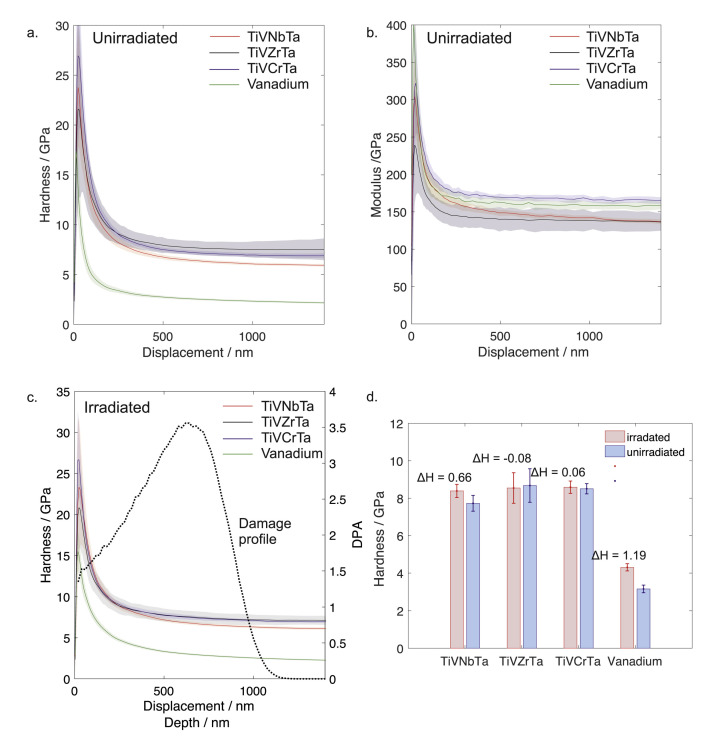
Indentation hardness and irradiation hardening plots (**a**) unirradiated indentation hardness vs. depth (**b**) Unirradiated indentation modulus vs. depth (**c**) Irradiated indentation hardness vs. depth. The irradiation damage profile is given by the dashed line. (**d**) Indentation hardness of irradiated and unirradiated HEAs and control sample at 300 nm indentation depth. Shaded area in (**a**–**c**) and error bars in (**d**) represent the standard deviation of 25 indentations. Reproduced from Reference [20].

A number of investigations have reported no or very limited irradiation hardening of BCC refractory-based concentrated alloys, even after damage to many dpas [20,22,24,30,129]. For instance, Moschetti et al. [129] found a small increase (∼10%) in nanohardness, yield strength and ultimate-tensile strength for He-irradiated TiZrNbHfTa. Interestingly, they also found that there was no loss of ductility following irradiation, suggesting good damage tolerance (although the alloy was in a nanocrystalline condition to allow for improved small-scale testing). Some have even found significant softening following irradiation [124]. Comparisons to pure refractory BCC metals irradiated under the same conditions again demonstrate the apparent improved resistance of concentrated multicomponent compositions, Figure 5. These trends need more explanation and investigation, and comparison, for example, to binary alloy combinations.

In contrast to BCC refractory HEAs, in CrFeNi-based alloys there has been evidence of significant hardening, which has been found to be similar to that in 316 stainless steel [78,79]. It is clear, then, that not all HEAs are guaranteed to have resistance to irradiation hardening. Zhao [130] suggests that the disparity in the responses of BCC vs. FCC HEAs may be due to the distribution of migration energies for vacancies and interstitials exhibiting a much larger overlap in BCC HEAs compared to FCC HEAs, leading to enhanced defect recombination.

### 4.5. Current Limitations of Experimental Studies

The limitations of much of the experimental work assessing HEA phase stabilities under irradiation has been highlighted summarised by Kumar et al. [78]. They noted in 2016 that many studies failed to assess alloys under conditions that would appropriately probe their resistance to irradiation damage, and this has, unfortunately, continued in more recent years. For instance, many studies have examined the irradiation of thin films, where defects can readily annihilate at surface, meaning that the damage will be artificially lower than it would be in the bulk (see recent useful analysis by Zinkle and Snead [131] for avoiding this pitfall). Others use of nanocrystalline materials, which comprise many boundaries acting as defect sinks. Some use irradiations at room T, where defect mobility is significantly inhibited, or irradiate with electrons, which do not cause the same damage as ions or neutrons. As noted by Kumar et al. [78], phase stability during irradiation typically involves a competition between the ballistic-induced dissolution of precipitate embryos, and the radiation-enhanced diffusion that leads to precipitate nucleation and growth. Hence, not providing sufficient thermal energy, or not replicating the nature of the irradiation damage or the influence of defect sinks, makes it difficult to assess what the true effects of HEA compositional complexity are on phase stability (or indeed other irradiation behaviour) [3,78].

As highlighted in the previous section, assessments of irradiation phase stability should be wary of the thermal stability of HEAs. Given the propensity of HEAs to decompose into multiple phases, often intermetallics, on annealing [108,112], care must be taken to ensure that phase decompositions are not simply a result of thermal effects.

Another key limitation of many studies to date is that they have rarely compared their results to conventional alloys or less compositionally-complex alloys (e.g., concentrated binary alloys comprising the same elements) assessed under the same conditions. This makes it difficult to ascertain whether the behaviour observed is truly exceptional. When comparisons have been made, the behaviour has not always been markedly different (or markedly improved) [78,110,113]. For instance, the dose needed for damage saturation in an Fe-Ni-Mn-Cr alloy was found to be comparable to that in more conventional Fe-Cr-Ni austenitic alloys at 500 °C [78]. In another example, the microstructural evolution and irradiation hardening in some FCC HEAs was found to be similar to 316H stainless steel at 300 °C, which led the authors to question the influence of HEA compositions and configurational entropy [113].

### 4.6. Summary

At present, the picture with regards to the irradiation damage resistance of HEAs is unclear, and it is uncertain whether or not HEAs possess any special resistance to irradiation damage *in general*. It seems unlikely that the influence of diffusion and lattice distortion on irradiation damage are any more prevalent than in more conventional alloys (i.e., the diffusion is unlikely to be anomalously slow, nor the lattice distortion high).

Nevertheless, there are two effects that the compositional complexities of HEAs might uniquely enable/enhance, and that warrant further investigation. First, their concentrated compositions are more likely to result in poor thermal conductivities, and this may affect the short-term damage evolution immediately following the cascade (potentially reducing the number of FPs formed, for instance). See, for example, the results of Reference [132]. Second, the compositional complexities of HEAs are more likely to deliver a spread in defect formation energies, and there is an indication that vacancy formation energies may be higher on average in comparison to less complex alloys. This could significantly influence the irradiation behaviour observed.

Of course, when considering HEAs there is always the possibility that exceptional properties will arise for some compositions that are not observed in general, as has been found for mechanical properties, for example, Reference [133]. They should not be written off entirely for this reason alone, but equally should not be referred to as exceptional in general terms if no universal trends are seen.

## 5. Challenges and Opportunities for HEA Development

There are a number of challenges, but also opportunities, that are afforded by the compositional space HEAs provide access to. The general design challenges associated with HEAs for engineering applications have been discussed elsewhere, for example, References [112,134], but here we focus on a few challenges and opportunities that are particularly relevant to nuclear applications and the implementation of HEAs in them: elemental selection for irradiation resistance and phase stability, problems with impurities, and modelling challenges.

### 5.1. Element Selection

Element selection is a challenge for all HEA alloy design efforts, because the compositional space is so vast and often encompasses areas that we know very little about. The elemental restrictions highlighted in Section 3 will restrict the options for many nuclear applications, but the number of options remains daunting. This presents both challenges and also an opportunities. Examples of each are discussed as follows.

#### 5.1.1. Opportunity—Tuning Compositions for Irradiation Response

It would be compelling if we were able to use the enhanced design space afforded by HEA compositions to *design in* irradiation resistance by optimising their compositions. For example, Zinkle and Snead [41] highlighted three options for improving the irradiation resistance of alloys to high dpa levels at high temperatures: (i) the utilisation of a matrix with inherent radiation tolerance, (ii) the selection of materials in which vacancies are immobile at the design operating temperatures, and (iii) the selection of materials with high sink strengths and densities for point defect recombination (with biases set accordingly). It is not practical for most metallic alloys to meet option (ii) at service temperatures [41], but the design of HEAs for nuclear could be guided by (i) and (iii), and the compositional freedom associated with HEAs is a significant opportunity.

A number of HEA studies have discussed the potential of tuning HEA irradiation properties by changing compositions [3,50,75,80,85,88,117,135]. For instance, in relation to a more inherently resistant matrix, Caro et al. [50] used ab-initio methods to investigate how composition modified the scattering mechanisms that control energy transport in the phonon subsystem, and discussed how it could be tailored for maximum scattering. In relation to sink strengths and densities, many HEA design strategies (e.g., as discussed in the following section) involve the introduction of controlled precipitation, and there is no reason to suppose that this could not be tailored to introduced sink strength optimisation. To this end, it is likely that investigations of how radiation-induced segregation influence the precipitation will be needed [136]. Significant challenges are associated with these design strategies, such as the need for complex models that predict the properties of multicomponent space. Nevertheless, the greater compositional freedom of HEAs is also an exciting prospect.

#### 5.1.2. Challenge—Avoiding Detrimental Phases

Much of the wider HEA literature has focussed on the pursuit of alloy compositions that form stable solid-solution microstructures. There are good reasons to suppose that such a pursuit should be part of HEA alloy design for nuclear applications, at least in the first instance. Irradiation damage in alloys that comprise significant fractions of multiple phases has often been found to result in preferential damage (including amorphisation) or precipitation of one phase, resulting in mechanical property degradation. It may only be tolerable if the precipitating phase is also a solid solution of the same crystal structure [124].

Following identification of stable solid solutions, the introduction of precipitates should then be addressed. The presence of at least some precipitates is typically essential for strengthening, especially at high temperatures, and the vast majority of engineering structural alloys, including those used in nuclear applications, contain fine precipitates that enhance properties. Furthermore, the introduction of a fine second phase dispersion can provide defect sinks as discussed above [41]. However, it is critical that the amount and type of precipitation is carefully controlled.

Examining the low-activation elements in Figure 2, it becomes apparent that the many of the HEAs formed by combinations of the low-activation transition metals will result in alloys based on BCC refractory elements. Examining these potential alloys in more detail, it can be observed that there will be a strong propensity to form Laves phases and sigma phase, as has been found in a number of studies of concentrated alloys comprising Ti, V, Cr, Mn, Fe, Ta and W [17,20,137]. Predicting the formation of these embrittling phases, and tailoring compositions to suppress their formation, remain challenges.

### 5.2. The Impurity Problem

Even if detrimental IM compounds are removed and favourable precipitates introduced in a controlled manner, the introduction of impurity elements, whether in production or in service, is likely to prove problematic for BCC HEAs. Key concerns include: (i) hydrogen embrittlement and retention, (ii) the embrittlement of refractory alloys by interstitial elements, and (iii) the presence of highly-activating trace elements in low-activation materials.

#### 5.2.1. Hydrogen Embrittlement and Retention

Only a few studies have examined the susceptibility of HEAs to hydrogen embrittlement, and there are no clear trends. CrFeMnCoNi has been found to be more resistant to hydrogen embrittlement than two conventional austenitic stainless steels (304 and 316 L), despite retaining more hydrogen than the other alloys and possessing a higher yield stress (which did not change on hydrogen charging). After gaseous charging, its ductility was found to decrease only marginally [138]. Conversely, CrFeMnCoNi with carbon additions was shown to exhibit a much larger ductility loss after charging [139], despite a lower overall hydrogen concentration than the CrFeMnCoNi studied in Reference [138].

An assessment of hydrogen permeation in CrFeMnCoNi has suggested similar behaviour to 316 L stainless steel [140]. Modelling work, on the other hand, has suggested that sluggish diffusion of H owing to a complex interstitial environment in HEAs (FeCuCrMnMo was assessed) should lead to reduced permeation rates [141]. Hydrogen retention in CrFeMnCoNi has been found to be higher than for 304 and 316 L steels [138], and far higher than in RAFM steel [140] (although this is almost certainly owing to the greater solubility afforded by its FCC structure in comparison to the BCC steel).

Hydrogen embrittlement and retention are likely to be more problematic in BCC HEAs than in HEAs, particularly in BCC refractory HEAs. It is well known that the mobilities of interstitial elements are typically higher in BCC metals than their FCC counterparts. BCC refractory alloys can also have considerable solubility for H, to the point that some BCC refractory alloys are being designed specifically for H storage, for example, References [142,143,144].

An interesting possibility regarding the hydrogen retention of HEAs may be afforded by the variable local atomic environments that a hydrogen atom will encounter in their structures. Any dissolved hydrogen will be released at a temperature that is dependent on the local environment of its trap site, meaning that an HEA may leak hydrogen over a wider envelope of temperatures rather than decompose over a narrow temperature range.

It has been highlighted that some efforts to suppress irradiation damage may in turn hamper efforts to limit tritium retention [41]. For instance, the boundaries of fine precipitates used as defect sinks are also commonly introduced into alloys for hydrogen trapping [145]. It is also known that irradiation-induced defects can themselves promote the trapping of tritium, meaning it increases with irradiation [146,147,148,149,150].

#### 5.2.2. Interstitials in BCC Refractory Alloys

The brittleness of HEAs based on BCC refractory elements is a potential show-stopping problem. Most refractory metals have high DBTTs in their pure form, and although efforts have been made to ‘ductilise’ some of them (for instance, adding Re to W [151]), the issue remains endemic and in most cases BCC refractory metals are found to be hard and brittle at room temperature. It is not surprising, therefore, that BCC refractory HEAs have typically been found to have high hardnesses in comparison to most conventional alloys, for example, References [16,19,20,21,24,28,31,124], and the results of tensile tests are very rarely reported.

It is well known that increased levels of interstitial elements C, N and O only increase brittleness, and the significant solubility of these elements in refractory BCC metals is relatively high, making them difficult to remove. Interstitial impurities can also influence irradiation responses. For instance, higher levels of interstitial impurities in BCC refractory metals have been found to result in enhanced void swelling [152]. Unfortunately, interstitial contamination can easily be introduced during primary processing, such as arc melting, or spark plasma sintering [17,18,153]. Further contamination can be introduced during heat treatment, even if samples are appropriately wrapped and encapulated in evacuated tubes. Ideally, concentrations should always be measured (e.g., using LECO) and reported accordingly, but the practice is relatively rare at present. Nevertheless, even if impurity levels are reported, a problematic reality exists—it is likely to be the interstitial content *in solution in the matrix* that has the most significant effect on properties, and this is not at all trivial to measure if corresponding precipitates or inclusions are also present.

In addition to their effect on mechanical and irradiation properties, it is also very likely that many of the refractory BCC HEA microstructures that have been reported have been influenced by interstitials. For instance, it is apparent that only small amounts of contamination are required in alloys containing Zr and Hf (amongst others) for compounds based on the rocksalt structure, for example, ZrC, to form, owing to the high anion vacancy concentration permitted [154,155]. Although small amounts of stable carbide/nitride/oxide may prove beneficial for irradiation resistance, uncontrolled and excessive levels will not, particularly if they coincide with high interstitial matrix concentrations.

#### 5.2.3. Guaranteeing Low Activation

It has been recognised that unavoidable impurities in alloying elements may mean that guaranteeing low activation in alloys, including HEAs, is difficult. Even a low-activation steel like EUROFER97 is exceptionally tricky to produce without introducing traces of highly-activating elements like Nb, which are derived from the primary ores and are difficult to remove during manufacture [15,156]. One might imagine that in HEAs, with high concentrations of many different elements each with their own impurities, the problem is likely to be exacerbated.

### 5.3. Modelling Challenges

In order to predict the irradiation response of HEAs, and hence design more radiation-resistant alloys, it will be necessary to generate advanced atomic-scale models of their behaviour. However, there are significant challenges associated with this. Atomic-scale modelling of HEAs and other highly-alloyed systems has been a challenge for the atomic scale modelling community since its early days. The manner in which to treat a solute species is typically more easy to compute at low concentrations, treating the solute as a substitutional point defect at low concentrations (or at the dilute limit) removes a number of the steric issues whilst binding energies and other configurational arrangements are deemed insignificant. Examples of where these methods have been used well include understanding the solubility of transmutation products in tungsten and zirconium (where extrinsic concentrations are low in most alloys).

A number of methods have been developed to account for the random or near-random arrangement of species in high alloy materials and have been subsequently applied in HEA systems:

**Partial occupancy mean field approach/coherent potential approximation** methods have been used whereby each atomic site is shared as a proportion between one type of atom and another. A simple BCC high entropy alloy consisting of MoNbTiTaW could be modelled as having an occupancy factor of 0.2 (when the HEA is equiatomic) and scaling the potential parameters appropriately. This has been used successfully in both classical modelling and density functional theory (DFT) applications. Early work by Körmann et al. [157] modelling the CoCrFeNiPd system and others to predict their magnetic properties including the Curie temperature, were used to great effect. The drawbacks of this methodology are that the complexity and local ordering/environment effects are difficult to differentiate. Partitioning of defects is also less descriptive and when considering radiation damage events, such as Frenkel formation energies, the specific behaviour of each species is distinct from one another. The benefits are that it is relatively computational inexpensive compared to the following methodologies (whether using DFT or classical atomic scale modelling methods).

**Random occupancy** methods have been used for HEAs quite successfully in the past and can be the most representative manner in modelling HEAs. The random nature of the species can be captured well through this approach and DFT modelling results through this methodology have successfully predicted phase stability of single phase high entropy alloys [158], partitioning of species [116], sub-lattice ordering [10,159] and solution/accommodation of other extrinsic defects (such as fission products [9]). The method can capture a spectrum of completely random structures to partially ordered structures and ascertain specific component to component bonding and their impact on the electronic structure of the material. The major drawback to this method is the relative computational expense. Each composition must be repeated at random until the statistics from the results are acceptable (sometimes >20 of each composition—some DFT cells that take many hours to complete may be prohibitive to compute).

**Classical modelling** methods, building systems using a randomly occupied lattice, have been used in good effect (e.g., References [160,161,162]). These less descriptive methods have the benefit of being able to interrogate larger features such as dislocations and surface effects as well as phenomena that typically take longer to simulate on the atomic scale such as quenching (rates are typically far higher than in experiments due to computational costs) and mechanical deformation studies requiring large system sizes (e.g., Reference [163]) although they require robust interatomic potentials to be predictive.

**Monte-Carlo methods and special quasi random structures (SQS)** are extremely robust methods for modelling these random alloys when the computational system size is limited (e.g., DFT, where typically 100–200 atoms are considered). The SQS methodology has been used successfully by a number of groups (e.g., References [164,165]). Special-quasi random structures [166] are optimised through methods such as Monte Carlo annealing to give the most random arrangement of atoms possible in a finite system (optimising the cluster vector). The results are useful to understand most phenomena in HEA systems (defect, thermal properties and structural stability) and is often considered the best route for initial modelling of HEA systems. Some small drawbacks include the difficulty of capturing local ordering/clustering effects but many of these issues can be designed to be included in the structural determination.

#### 5.3.1. Particular Considerations

Previously, it was noted that point defects in alloys such as HEAs cannot be represented by a single formation energy [9,116], rather because of the variation in the local coordination and symmetry considerations, a range of energies for vacancies, interstitials and substitutional defects result in a range of solution, Schottky, Frenkel and anti-site energies. In addition to point defect computations, DFT calculations that involve the computation of phonons (e.g., to predict thermal properties such as thermal expansion) are more complicated in HEAs [167] compared to simpler systems due to the symmetry considerations that can be exploited in simple systems that vastly reduce the computational expense of methods such as the quasi-harmonic and harmonic approximations (e.g., Reference [168], which also captures electronic effects related to thermal and phonon properties). Other methods can be used instead (including molecular dynamics methods [169,170]), but these are not necessarily more computationally efficient or robust when combined with DFT, providing a significant hurdle to modelling HEAs. As already noted, classical atomic scale modelling methods are less constrained computationally, however, interatomic potentials that are robust across significant temperature scales are not abundant, especially in these compositionally complex alloys.

To end on a positive note in this regard: it should be noted that as the phonons are likely to be scattered far more readily within a HEA compared to a simpler crystalline system. The active phonon modes are likely to be shorter than in other alloys due to an increase in mass-difference scattering [171]. Therefore, smaller systems are likely to be able to represent the physical behaviour of the materials (although care should be taken in order to properly assess the impact of system size).

#### 5.3.2. Application to Alloy Design

The combinatorial complexity of HEAs means that computational methods can be considered necessary to navigate the plethora of systems. Several attempts have been made (e.g., References [172,173,174,175]), combining thermodynamic approaches, empirical observations and other methods including atomic scale simulation and CALPHAD [176,177] methods.

A robust example of one of these tools is the alloyASAP method developed by King et al. [158] focusing on single phase HEAs that uses two key parameters: the regularly utilized δ (a representation of the difference in atomic radii of the constituent elements. See Singh et al. [178]) with a parameter Φ, which is the ratio of the free energy of the system in perfect solid solution to the lowest free energy of a constituent binary system of the alloy in question. These values are predicable using a combination of thermodynamic modelling (e.g., via DFT) and experimental methods. The tools available predict HEA thermal stability, melting point and a range of other properties (including thermal neutron cross section) have been used successfully, for example, References [28,31,179]. The methodology could be adapted and modified readily to capture other phenomena not presently considered.

## 6. Summary and Conclusions

- Our understanding of the irradiation responses of HEAs remains in its infancy, and much work is needed in order for our knowledge of any single HEA system to match our understanding of conventional alloys like austenitic steels. Nevertheless, the expanded compositional freedom afforded by HEAs represents a unique opportunity for the design of alloys for advanced nuclear applications. In particular, the tuning of compositions over a large range to optimise particular irradiation responses could be very powerful.
- Elemental palettes may be limited by the restrictions imposed in certain nuclear environments (e.g., the need for low activation or low neutron cross section), but there remains a vast unexplored compositional space to probe.
- Many authors have claimed that the sluggish diffusion of defects in HEAs, as well as lattice distortion, make them more irradiation resistant. The evidence in support of this generalisation is unconvincing. Nevertheless, there may be some effects that are uniquely enhanced in HEAs—for example, their lower conductivities may change the cascade dynamics and deliver fewer defects for each cascade. Also, the apparent limited irradiation hardening of BCC refractory HEAs also warrants further exploration.
- It is evident that many studies have reported HEA irradiation properties under conditions that are unlikely to reproduce their response in bulk form (e.g., through the irradiation of thin films, or by the use of material in nanostructured form). Furthermore, there has been limited comparison to conventional alloys studied under the same conditions, making it difficult to assess their true potentials.
- It is fortuitous that most of the low-activation elements that are required in fusion applications also form BCC structures, which are generally thought to possess improved swelling resistance versus FCC alloys (although this remains a matter of debate). Nevertheless, there are considerable challenges to the development of BCC refractory HEAs, such as low DBTT temperatures, embrittlement from impurities and H and He, and high solubilities for H (tritium retention in fusion applications will likely be very problematic) and other interstitial elements.
- Atomic scale modelling methods and computing resources are in an ideal state to advance the mechanistic understanding of HEAs in order to accelerate development and licensing of HEAs for use in nuclear applications. Mechanistic models can accelerate licensing through efficient use of otherwise expensive integral test reactor experiment results combined with separate effects experiments. Mechanistic models also guide experimental design and aid the comprehension of experimental findings.

## 7. Remaining Questions

Some key remaining questions, which researchers may wish to direct their attention to, include:

- Do HEAs possess enhanced intrinsic damage resistance versus conventional alloys? There is little indication that sluggish diffusion is likely to be significant in general, but are there changes in cascade dynamics and defect energetics brought about by HEA compositional complexities that are beneficial? For instance, it has been suggested that the reduced thermal conductivities generally possessed by HEAs may prolong the thermal spike and change the size and shape of the cascade, leading to more opportunity for defect recombination. Is this generally true for all HEAs? This deserves more attention.
- Do refractory BCC HEAs possess special irradiation damage resistance, versus less-concentrated BCC alloys and/or FCC HEAs? If so, what is the mechanism for this? There has been some suggestion that the defect energetics in some BCC refractory HEAs may be conducive to superior irradiation damage resistance, but further confirmation of whether this true more generally is needed.
- How can we best design HEAs for nuclear applications? Designing HEAs for enhanced irradiation tolerance is still in its infancy, and there’s not yet been a study that’s sought to tune, for example, the defect formation energies, or sink strengths or biases, through the optimisation of HEA compositions. Design efforts will almost certainly require the use of advanced atomic-scale models to predict irradiation response, and there remain challenges associated with their construction.
